# New Amides and Phosphoramidates Containing Selenium: Studies on Their Cytotoxicity and Antioxidant Activities in Breast Cancer

**DOI:** 10.3390/antiox10040590

**Published:** 2021-04-11

**Authors:** Mikel Etxebeste-Mitxeltorena, Daniel Plano, Nora Astrain-Redín, Cristina Morán-Serradilla, Carlos Aydillo, Ignacio Encío, Esther Moreno, Socorro Espuelas, Carmen Sanmartín

**Affiliations:** 1 Department of Pharmaceutical Technology and Chemistry, University of Navarra, Irunlarrea 1, E-31008 Pamplona, Spain; metxebeste@alumni.unav.es (M.E.-M.); dplano@unav.es (D.P.); nastrain@alumni.unav.es (N.A.-R.); cmoran.3@alumni.unav.es (C.M.-S.); caydillo@unav.es (C.A.); emorenoa@unav.es (E.M.); sespuelas@unav.es (S.E.); 2 The Navarra Medical Research Institute (IdiSNA), Irunlarrea 3, 31008 Pamplona, Spain; 3 Tropical Health Institute of the University of Navarra (ISTUN), University of Navarra, Irunlarrea 1, E-31008 Pamplona, Spain; ignacio.encio@unavarra.es; 4 Department of Health Sciences, Public University of Navarra, Avda. Barañain s/n, 31008 Pamplona, Spain

**Keywords:** amide, cytotoxicity, diselenide, phosphoramidate, selenocyanate

## Abstract

Breast cancer is a multifactor disease, and many drug combination therapies are applied for its treatment. Selenium derivatives represent a promising potential anti-breast cancer treatment. This study reports the cytotoxic activity of forty-one amides and phosphoramidates containing selenium against five cancer cell lines (MCF-7, CCRF-CEM, HT-29, HTB-54 and PC-3) and two nonmalignant cell lines (184B5 and BEAS-2B). MCF-7 cells were the most sensitive and the selenoamides I.1f and I.2f and the selenium phosphoramidate II.2d, with GI50 values ranging from 0.08 to 0.93 µM, were chosen for further studies. Additionally, radical scavenging activity for all the compounds was determined using DPPH and ABTS colorimetric assays. Phosphoramidates turned out to be inactive as radical scavengers. No correlation was observed for the antioxidant activity and the cytotoxic effect, except for compounds I.1e and I.2f, which showed dual antioxidant and antitumor activity. The type of programmed cell death and cell cycle arrest were determined, and the results provided evidence that I.1f and I.2f induced cell death via autophagy, while the derivative II.2d provoked apoptosis. In addition, Western blot analysis corroborated these mechanisms with an increase in Beclin1 and LC3-IIB and reduced SQSTM1/p62 levels for I.1f and I.2f, as well as an increase in BAX, p21 and p53 accompanied by a decrease in BCL-2 levels for derivative II.2d.

## 1. Introduction

Cancer is considered a serious public health burden because it affects millions of people worldwide. Cancer is the second lethal disease globally, and about one out of six deaths are due to cancer [1]. Worldwide, breast cancer is one of the most common cancers, the fifth most prevalent cause of cancer death, and the main cause of cancer death in women [2]. Although many novel small molecules with cytotoxic activity toward breast cancer have been designed for the last decade, it is meaningful to develop more effective and safer drugs for this illness.

In this context, researchers worldwide have focused their attention on the synthesis of selenium-containing compounds due to their promising results against different types of cancer [3]. Several chemical entities in selenocompounds have demonstrated potent inhibitory effects on cell proliferation, mainly by interfering in the redox homeostasis and cell signaling of cancer cells [4]. Among the different mechanisms implicated, apoptosis, autophagy, DNA damage and HDAC inhibition are among the most studied [5]. Other implicated mechanisms could be the modulation of the oxidative stress, antioxidant effects through selenoproteins, the modulation of some kinase activity, the inhibition of the mTOR regulatory cascade or a combination of some of those described [6,7,8]. Moreover, different studies have described organoselenium derivatives with potent activity against triple-negative breast cancer, i.e., a type of breast cancer that lacks estrogen, progesterone and HER2 receptors [9,10], or as adjuvants against resistant breast cancer, i.e., a doxorubicin-resistant subline overexpressing ABCB1 derived from MCF-7 cells [11]. In addition, small molecules containing selenium such as methylseleninic acid suppressed breast cancer growth via the JAK2/STAT3 pathway [12].

In spite of the controversy about the use of antioxidants for cancer treatment, different clinical trials have demonstrated their efficacy [13]. If we focus on breast cancer, considering the existence of many subtypes with different redox status, several compounds such as *N*-acetylcysteine [14], garlic derivatives [15] or flavonoids [16] have been reported for their specific properties against breast cancer. Moreover, the association between total selenium content in the body and breast cancer incidence has been studied extensively [17,18]. In this context, different selenium derivatives have been described with this purpose. 

Accumulating evidence in the literature has illustrated that among the different selenated scaffolds, selenenocyanate [19,20] and diselenide [21,22,23] fragments are important pharmacophores that significantly suppress breast cancer. Furthermore, our research group has reported that selenocyanate and diselenide entities possess potent antitumor activity [24,25,26,27]. Consequently, these motifs are considered as an encouraging template for designing a new category of selenium compounds. Taking as prototypes two effective analogs, 4-aminophenylselenocyanate and bis(4-aminophenyl)diselenide, we design a new generation of derivatives with amide and phosphoramidate linkages and diverse alkyl, aryl and heteroaryl substitutional units. The amide link was used because amides assume distinct conformations and are present in many anticancer drugs [28]. The inclusion of phosphoramide was based on its presence in many antitumoral compounds such as cyclofosfamide, ifosfamide, trofosfamide, perfosfamide and evofosfamide [29]. 

In this study, we provide an evaluation of the synthesized selenium derivatives as cytotoxic and antioxidant agents. The antiproliferative effect was tested in vitro using five human cancer cell lines as well as two nonmalignant cell lines. The antioxidant activity was determined by DPPH and ABTS assays. To better understand the potential mechanisms of action, the most active and selective compounds were further evaluated for their effect on the cell cycle distribution, cell death induction and alteration of different proteins related to autophagy or apoptosis processes.

## 2. Materials and Methods

### 2.1. Chemistry

Synthesis, purification and characterization of the compounds have been previously described [30]. Chemicals were purchased from E. Merck (Darmstadt, Germany), Panreac Química S.A: (Montcada I Reixac, Barcelona, Spain), Sigma-Aldrich Química S.A. (Alcobendas, Madrid, Spain) and Across Organics (Janssen Pharmaceuticals, Geel, Belgium).

### 2.2. Biological Evaluation

#### 2.2.1. Cell Cultures

Cell lines were provided by the European Collection of Cell Cultures (ECACC) or by the American Type Culture Collection (ATCC). Seven cell lines were used: MCF-7 (breast adenocarnicoma), 184B5 (nonmalignant, mammary gland derived), CCRF-CEM (lymphoblastic leukemia), HT-29 (colon carcinoma), HTB-54 (lung carcinoma), BEAS-2B (nonmalignant, derived from the bronchial epithelium) and PC-3 (prostate carcinoma). MCF-7, CCRF-CEM, HT-29, HTB-54, BEAS-2B and PC-3 cell lines were grown in RPMI medium (Gibco) supplemented with 10% fetal bovine serum (FBS; Gibco), 100 units/mL penicillin and 100 mg/mL streptomycin (Gibco). 184B5 cells were grown in DMEM/F12 medium supplemented with 5% FBS, 1 × ITS (Lonza), 100 nM hydrocortisone (Aldich), 2 mM sodium pyruvate (Lonza), 20 ng/mL EGF (Sigma-Aldrich), 0.3 nM trans-retinoic acid (Sigma-Aldrich), 100 units/mL penicillin and 100 mg/mL streptomycin. Cells were maintained at 37 °C and 5% CO_2_.

#### 2.2.2. Cytotoxic and Antiproliferative Activities 

The cytotoxic effect of each substance was tested by the MTT method. Each compound was initially dissolved in DMSO at a concentration of 0.01 M, and serial dilutions were prepared with nonsupplemented medium. The cytotoxic effect of each compound was tested at 50 and 10 µM as a first screening. Compounds with a cell growth percentage under 50% at 10 µM in at least one cell line were selected and tested at five different concentrations ranging between 0.01 and 100 µM.

A total of 1 × 10^4^ cells/well in 96-well plates were treated with increasing concentrations of the corresponding compounds for 48 h at 37 °C in a humidified atmosphere containing 5% CO_2_. Then, cells were incubated with 50 µL of MTT (2 mg/mL stock) for 4 h. The medium was then removed by aspiration, and the formazan crystals were dissolved in 150 mL of DMSO. Results are expressed as GI_50_, the concentration that reduces by 50% the growth of treated cells with respect to untreated controls (0.1% DMSO); TGI, the concentration that completely inhibits cell growth; and LC_50_, the concentration that kills 50% of the cells. Data were obtained from at least 3 independent experiments performed in quadruplicate. The standard error of the means (SEM) for the cytotoxic parameters was calculated applying the standard deviation formula to the mean values of each parameter for the three independent experiments performed.

#### 2.2.3. Evaluation of Cell Cycle Progression and Cell Death

For the MCF-7 cell line, both cell cycle analysis and the cell death percentage were determined using the Apo-Direct kit (BD Pharmingen) based on the TUNEL technique, according to the manufacturer’s instructions. Cells were seeded in 25 cm^2^ flasks (3 × 10^6^ for 24 h of treatment, 2 × 10^6^ for 48 h and 10^6^ for 72 h) treated with DMSO (negative control), 6 µM of camptothecin (positive control) and different concentrations of I.1f, I.2f and II.2d.

After treatment, cells were collected and fixed with 1% paraformaldehyde (Sigma) in PBS (pH = 7.4), incubated in ice for 40 min, collected by centrifugation, washed with PBS and incubated with 70% ethanol for 30 min at −20 °C. After fixation, cells were washed twice with PBS and incubated for 1 h at 37 °C with FITC dUTP-DNA Labeling Solution. Cells were then rinsed and incubated in the dark for 30 min at room temperature with PI/RNase staining buffer before being analyzed by flow cytometry (Coulter Epics XL, Beckman Coulter Flow cytometer). Then, cells were treated with compounds I.1f (80 µM), I.2f (30 µM) and II.2d (5 µM) at different times (from 8 to 72 h) following the same methodology.

For autophagy and caspase inhibition assays, cells were pretreated with 100 nM of autophagy inhibitor (wortmannin, Santa Cruz) and 50 µM of pan-caspase inhibitor (Z-VAD-FMK, BD Pharmingen) for 1 h. The cells were treated with the compounds I.2f (30 µM) and II.2d (20 µM) for 24 h and I.1f (80 µM) for 72 h. Samples were processed following the same methodology stated above. DMSO was used as a negative control for both inhibitors.

#### 2.2.4. Protein Analysis

After treatment, cells were lysed in cell lysis buffer consisting of: 20 mmol/L Tris-HCl (pH 7.5), 150 mmol/L NaCl, 1% Triton X-100, supplemented with protease inhibitors and phosphatase inhibitors (10 mmol/L sodium fluoride and 10 mmol/L sodium orthovanadate) for 30 min on ice. Lysates were centrifuged at 13,200× *g* for 15 min at 4 °C to remove cell debris. The nonprotein fraction and supernatants were stored at −80 °C before use. Protein concentration was determined with the BCA Protein Assay. Cell samples (20–40 μg) were placed in SDS-sample buffer and 2% 2-β-mercaptoethanol, boiled for 5 min and subjected to SDS-PAGE on 12% Tris-glycine gels. Separated proteins were transferred onto 0.22 μm nitrocellulose membranes at 100V for 1 h. The membranes were incubated in blocking solution (5% nonfat dry milk-TBS-Tween-20) for 1 h at room temperature. Primary specific antibodies were incubated in 5% milk-TBS-Tween-20 (1 h, room temperature) to detect LC3B, Beclin-1 (D40C5), SQSTM1/p62, AMPK, JNK, BCL-2, BAX, p-53 and p21 (Cell Signaling) and actin (Santa Cruz Biotechnology). After incubation with the HRP-conjugated secondary antibody (Cell Signaling) in 5% milk-TBS-Tween-20 (1 h, room temperature), a chemiluminescence kit was used for visualization.

#### 2.2.5. Antioxidant Activity

##### DPPH Radical Scavenging Assay

The DPPH (1,1-diphenyl-2-picrylhydrazyl radical) assay measures the hydrogen donation ability of the antioxidant to convert the stable DPPH free radical into 1,1-diphenyl-2-(2,4,6-trinitrophenyl)-hydrazine. After radical reaction with the compounds, a decrease in the absorbance was detected at 517 nm, which is accompanied by a change of color from violet to light-yellow. The target compounds were dissolved in methanol to make 1 mg/mL stock solutions, which were diluted to five concentrations as test samples. Ascorbic acid was used as a standard, and the experiment was carried out on 96-well plates. The DPPH solution was prepared at a 100 µM concentration. For each test, 100 µL of DPPH solution was added to 100 µL of methanolic solution containing the tested derivatives, and the absorbance was determined at different time points. All the measurements were carried out in triplicate. Results were expressed as the percentage of the radical scavenged, calculated using the following formula:

(1) $$\%\;\mathrm{DPPH}\;\mathrm{radical}\;\mathrm{scavenging}=\frac{A_{\mathrm{control}}\;-\;A_{\mathrm{sample}}}{A_{\mathrm{control}}}\;\times \;100$$

where A_control_ refers to the absorbance of the negative control, and A_sample_ refers to the absorbance of the tested compounds. Results are expressed as a percentage of DPPH radical scavenged ± SEM. 

##### ABTS Radical Scavenging Assay

ABTS radical scavenging was additionally assayed with a colorimetric assay following a previous methodology [24]. Briefly, ABTS was first dissolved in deionized water at a concentration of 1 mg/mL and then oxidized to ABTS^•+^ with potassium persulfate (2.45 mM final concentration). This cocktail was kept overnight away from light at room temperature. Then, this ABTS^•+^ reaction mixture was diluted with a 50% ethanolic solution in order to achieve absorbance values of 0.70 ± 0.02 at 734 nm for measurements. Finally, 1 mg/mL stock solutions for the selected derivatives were formed in absolute ethanol and 20 µL of these solutions were added to 180 µL of the diluted ABTS^•+^ cocktail. After 6 min of incubation, absorbances at 734 nm were registered. A 50% ethanolic solution was used as blank along with Trolox (TROL) and ascorbic acid (Asc) as positive controls. All of the determinations were performed in triplicate using 96-well plates. The same time intervals as in the DPPH assay were also measured. The ability to scavenge ABTS^•+^ was calculated using the following formula:

(2) $$\%\;\mathrm{ABTS}\;\mathrm{radical}\;\mathrm{scavenging}=\frac{A_{\mathrm{control}}\;-\;A_{\mathrm{sample}}}{A_{\mathrm{control}}}\;\times \;100$$

where A_control_ refers to the absorbance of the negative control and A_sample_ refers to the absorbance of the tested compounds. The results are expressed as percentage of ABTS radical scavenging ± SEM.

#### 2.2.6. Statistical Analysis

Data were expressed as the mean ± SEM and analyzed by the Student’s *t*-test. * Statistical significance was defined as *p* < 0.05 (*); *p* < 0.01 (**) or *p* < 0.001 (***).

## 3. Results

### 3.1. Chemistry

**Figure 1:**
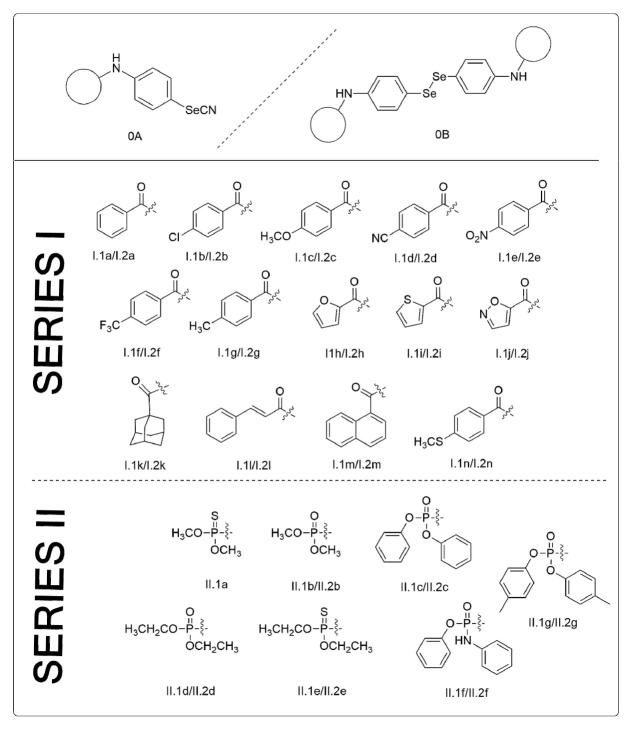
Chemical structures of series I (amides) and II (phosphoramidates). The diselenides derived from 0B were coded as I.1a-n and II.1a-f, and the selenocyanates obtained using 0A as starting material were coded as I.2a-n and II.2-f.

The route adopted for the synthesis of the novel forty-one compounds presented in this work has been previously reported [30,31]. Structures are summarized in Figure 1.

The synthesis of both series of compounds followed similar synthetic routes. Briefly, 4-aminophenylselenocyanate (0A) and bis-(4-aminophenyl)diselenide (0B) were achieved as previously reported [30]. For series I, selenocyanate derivatives (1a-1n) were obtained by the reaction of the amine 0A with the corresponding acid chlorides. Diselenides were synthesized by the reaction between 0B and the corresponding acid chloride (2d, 2k and 2m) or by the reduction of the corresponding selenocyanate analogs with sodium borohydride to yield 2a-2c, 2e-2j, 2i and 2n [30]. 

For series II, 0A and 0B were treated dropwise with the corresponding phosphoryl chlorides under different temperatures, atmospheric conditions and reaction times [31].

### 3.2. Biology

#### 3.2.1. Cytotoxicity and Antiproliferative Activities

**Figure 2:**
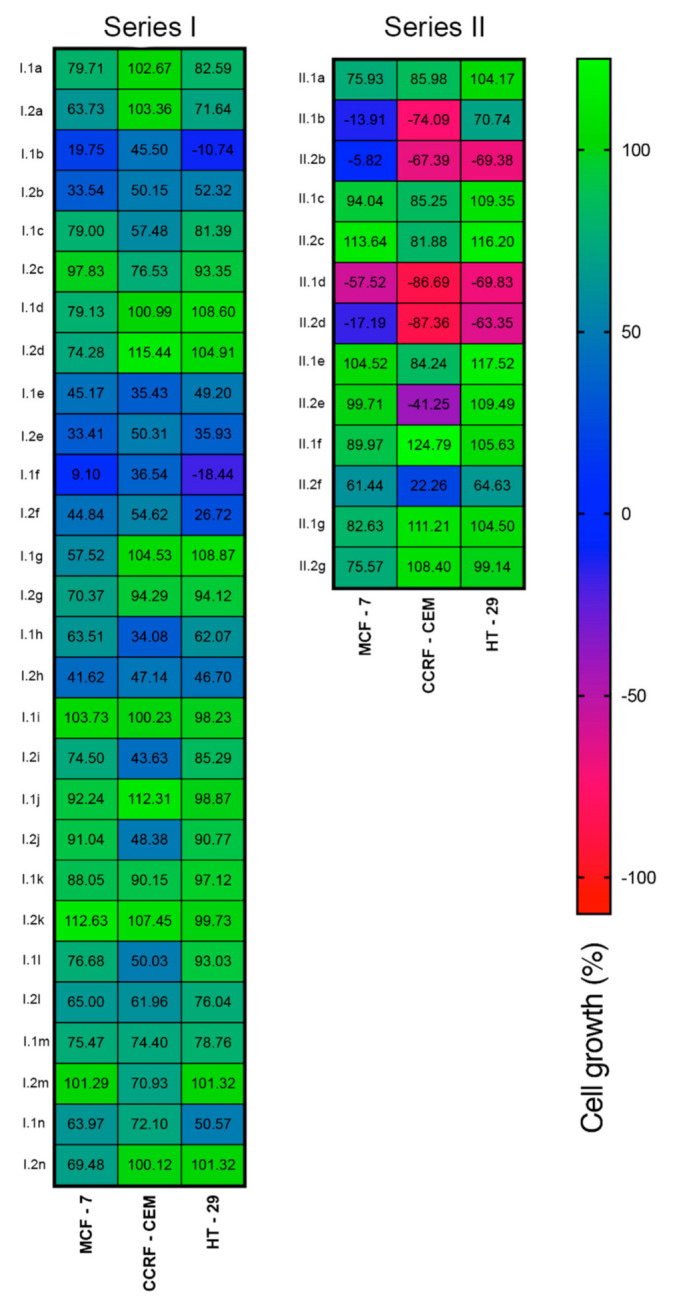
Cell growth percentage of MCF-7, CCRF-CEM and HT-29 cells treated with series I and II compounds at 10 µM after 48 h.

Herein, two series of compounds based on amides (series I) and phosphoramidates (series II) derived from 4-aminophenylselenocyanate and bis-(4-aminophenyldiselenide) were tested in vitro against cell lines derived from breast adenocarcinoma (MCF-7), lymphoblastic leukemia (CCRF-CEM) and colon adenocarcinoma (HT-29). Evaluation was performed at 48 h of treatment following the MTT (3-(4,5-dimethylthiazol-2-yl)-2,5-diphenyltetrazolium bromide) methodology as previously described [25]. The in vitro anticancer activity was determined using a two-stage process. The first stage involved the screening of all compounds at two doses (50 and 10 µM). The growth inhibition percentages obtained at 10 µM are shown in Figure 2.

Twelve compounds: 1b, 2b, 1e, 2e, 1f, 2f, 1h and 2h (series I) and compounds 1b, 2b, 1d and 2d (series II) showed cell growth below 50% at a 10 µM concentration. Hence, they were tested at five concentrations between 0.01 and 100 µM against five cancer cell lines, MCF-7, CCRF-CEM, HT-29, HTB-54 (lung carcinoma) and PC-3 (prostatic adenocarcinoma), and two nonmalignant cells, 184B5 and BEAS-2B. GI_50_, TGI and LC_50_ values were calculated from the curves and are shown in Table 1. In addition, selectivity indexes (SI) for tumor cells compared with nonmalignant ones were estimated according to the formulas GI_50_ (184B5)/GI_50_ (MCF-7) and GI_50_ (BEAS-2B)/GI_50_ (HTB-54) (Table 2). Cisplatin was used as a standard drug. It was found that MCF-7, CCRF-CEM and HT-29 cells were more sensitive to most derivatives in comparison to other cancer cells (HTB-54 and PC-3). In fact, MCF-7 presented the highest sensitivity with eleven derivatives (all of them with the exception of 1.1h) with GI_50_ < 10 µM. In general, different profiles were observed for amides and phosphoramidates. Thus, phosphoramidates II.1d, II.2b, and II.2d displayed a cytotoxic profile in MCF-7, CCRF-CEM and HT-29, whereas the amides only inhibited the growth in the same cells (I.1b, I.1f or I.2f). The presence of electron-withdrawing groups (-NO_2_, -CF_3_, -Cl) in the phenyl ring favored the cytotoxic activity in the amides, but in the phosphoramidates, the best results were obtained for aliphatic chains methoxy (II.1b, II.2b) and ethoxy (II.1d, II.2d). Additionally, the replacement of oxygen (II.1b, II.1d, II.2b, II.2d) by sulfur (II.1a, II.1e, II.2e) resulted in the abrogation of the activity. In relation to selenium entity, amides with selenocyanate motif were more active than the corresponding diselenides, and this is particularly evident in PC-3 (compounds 1.1b versus 1.2b, 1.1e versus 1.2e, 1.1f versus 1.2f, 1.1h versus 1.2h). However, no significant differences were observed for phosphoramidates. 

The lack of selectivity toward cancerous cells is one of the major issues during the discovery and development of new anticancer agents. Therefore, we evaluated the cytotoxic activity of our compounds against two cell lines derived from nonmalignant breast tissue (184B5) and no malignant bronchial epithelium (BEAS-2B) (Table 2). Data analysis showed that some of the compounds with potent anticancer activity also exhibited great cytotoxicity on normal cells (184B5 and BEAS-2B), demonstrating that some of them exhibited poor selectivity. However, derivatives I.1f, I.2e and I.2f, displayed SI higher than 10 in the breast, which is considered by the literature [33] as a threshold to be considered selective. Additionally, derivatives II.2b and II.2d presented SI values of 7.56 and 6.33 in the breast. If we focused on lung carcinoma, II.2d was the most selective with SI = 2.85.

Collectively, considering all the results, compounds I.1f, I.2f and II.2d showed the most significant cytotoxicity against all the screened cell lines along with acceptable safety and were picked for further pharmacological research in the breast adenocarcinoma cell line MCF-7.

#### 3.2.2. Radical Scavenging Activity

**Figure 3:**
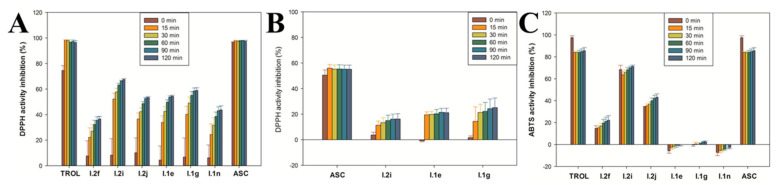
In vitro radical scavenging activity for the most active radical scavengers of series I using the colorimetric assays of DPPH ((**A**) for concentrations of 0.03 mg/mL and (**B**) for concentrations of 0.003 mg/mL) and ABTS (**C**).

In a first approach, the radical scavenging activity for all the synthesized compounds was characterized by the DPPH colorimetric assay at a dose of 0.03 mg/mL of each compound and six different time points (0, 15, 30, 60, 90 and 120 min). Ascorbic acid and Trolox were used as radical scavenger gold standards. Three diselenides (I.1e, I.1g and I.1n) and three selenocyanates (I.2f, I.2i and I.2j) from series I showed DPPH activity inhibition values greater than 35% after 30 min (Figure 3A) of treatment and were considered as radical scavengers. Surprisingly, no phosphormidates presented radical scavenging activity (data not shown). None of these six selenoderivatives reached DPPH activity inhibition values, as shown by the gold standards, but they can be considered potent radical scavengers, with compound I.2i being the most active radical scavenger of all the synthesized compounds. No correlation was found for either structure–radical scavenging activity or antitumor–radical scavenging activity, except for compound I.2f, one of the selected cytotoxic agents. To further confirm this antioxidant activity, three compounds (I.1e, I.1g and I.2i) were also evaluated as DPPH radical scavengers at a lower concentration of 0.003 mg/mL (Figure 3B). As expected, radical scavenging activity for these compounds was lower compared with the high dose, but still, some moderate effect was observed.

In a second approach, the antioxidant activity of the six compounds that showed DPPH activity inhibition was also further confirmed using the ABTS colorimetric assay (Figure 3C). Surprisingly, only the three selenocyanates also displayed ABTS activity inhibition capacity.

#### 3.2.3. Apoptosis and Cell Cycle Arrest

Apoptosis and cell cycle arrest are typical mechanisms of action for many anticancer drugs, including selenocompounds [34,35,36]. In a first approach, the effect of the selected compounds on cell cycle progression and the induction of apoptosis was studied in MCF-7 cell cultures. Analyses were performed by flow cytometry using the Apo-Direct kit, based on the TUNEL technique under the conditions described by the manufacturer. The cells were treated with the corresponding compound at different concentrations and time points. Camptothecin was used as a positive control at 6 µM.

**Figure 4:**
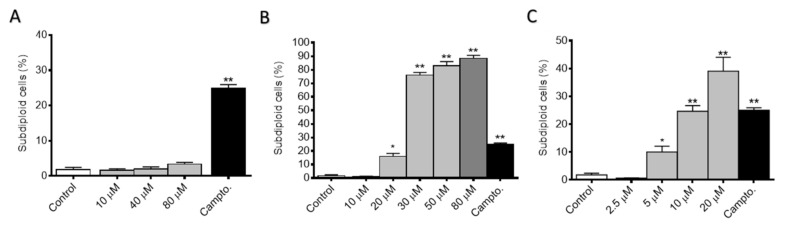
Percentage of subdiploid cells in MCF-7 cultures after 24 h of treatment with increasing doses of I.1f (**A**), I.2f (**B**) or II.2d (**C**). * Statistical significance with values of *p* < 0.05; ** statistical significance with values of *p* < 0.01.

**Figure 5:**
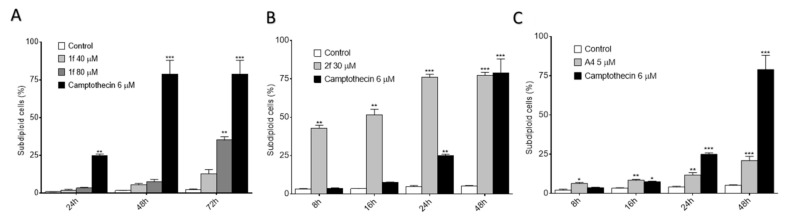
Percentage of subdiploid cells in MCF-7 cell cultures after treatment for the indicated time period with: 40 and 80 μM of I.1f (**A**), 30 μM of I.2f (**B**) and 5 μM of II.2d (**C**). * Statistical significance with values of *p* < 0.05; ** statistical significance with values of *p* < 0.01; *** statistical significance with values of *p* < 0.001.

As seen in Figure 4, compound I.1f did not stimulate cell death at 24 h when concentrations ranging from 10 to 80 µM were added, whereas derivatives I.2f and II.2d provoked a significant increase in the number of death cells (subdiploid cells) in a concentration-dependent manner. On the other hand, if we considered different time points and concentrations, all of them induced cell death in a time- and concentration-dependent manner (Figure 5).

**Figure 6:**
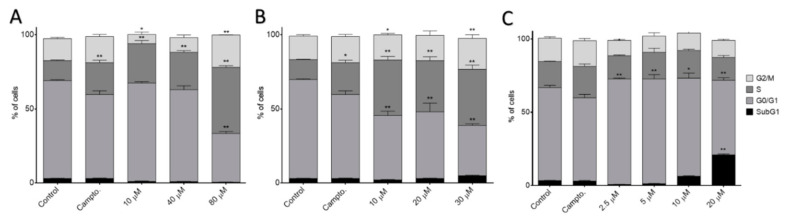
Cell cycle phase distribution of MCF-7 cell cultures after 24 h of treatment with different doses of I.1f (**A**), I.2f (**B**) and II.2d (**C**). * Statistical significance with values of *p* < 0.05; ** statistical significance with values of *p* < 0.01.

**Figure 7:**
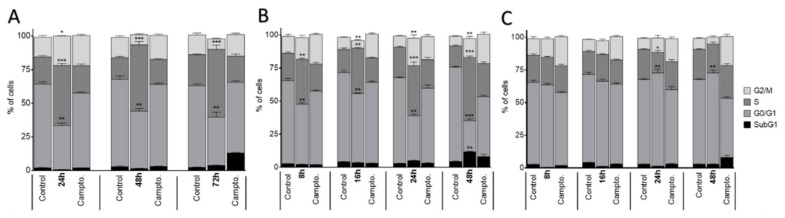
Cell cycle phase distribution of MCF-7 cell cultures treated for different time periods with I.1f (80 μM) (**A**), I.2f (30 μM) (**B**) and II.2d (5 μM) (**C**). * Statistical significance with values of *p <* 0.05; ** statistical significance with values of *p* < 0.01; *** statistical significance with values of *p* < 0.001.

To further understand the mechanism of action of these potent compounds, their effects on cell cycle distribution in MCF-7 were studied by flow cytometry. This assay was carried out with different concentrations (Figure 6) and different time points (Figure 7). The amides I.1f and I.2f increased the number of cells in the S phase dose-dependently, and the phosphoramidate II.2d arrested them in G0/G1 phase after 24 h of treatment (Figure 6).

After treatment with I.1f, I.2f and II.2d at various time points (24–72 h for I.1f and 8–48 h for I.2f and II2d), the results suggested that these compounds arrested cell cycle in a time-dependent fashion (Figure 7).

#### 3.2.4. Compounds I.1f and I.2f Induce Autophagy-Mediated Cell Death and Compound II.2d Caspase-Mediated Cell Death

Apoptosis and autophagy are considered as two recognized pathways for anticancer agents due to their effects on cell survival [37,38]. In the next step, in order to investigate whether the cell death pathway is related to apoptosis or autophagy, we explored the effect of pre-treated cells with either an autophagy inhibitor (wortmannin) or a pan-caspase inhibitor (Z-VAD-FMK). According to the previous results, determinations were performed after treatment with 80 μM of I.1f for 72 h, 30 μM of I.2f for 24 h and 20 μM of II.2d for 24 h.

**Figure 8:**
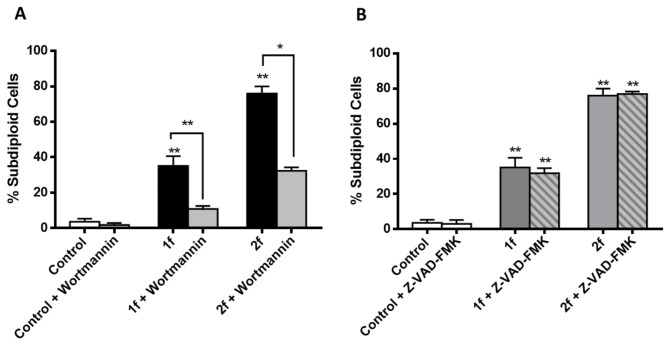
Cell death induced by compounds I.1f and I.2f blocked by wortmannin (**A**) but not by pan-caspase inhibitor Z-VAD-FMK (**B**). * Statistical significance with values of *p* < 0.05; ** statistical significance with values of *p* < 0.01.

As illustrated in Figure 8, preincubation with wortmannin prevented cell death induced by both amides I.1f and I.2f, while preincubation with Z-VAD-FMK did not. These data suggested that autophagy is implicated in the cell death induced by these two compounds.

**Figure 9:**
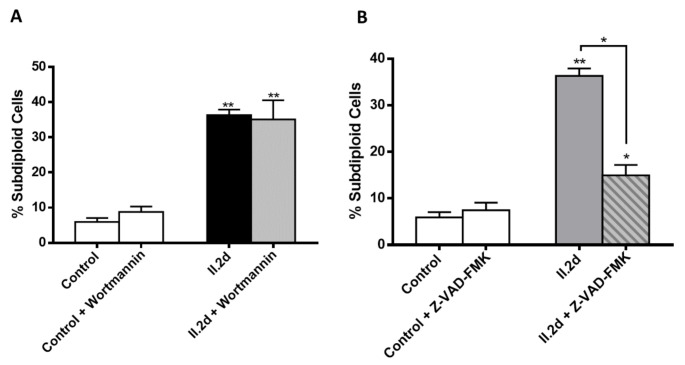
Z-VAD-FMK (**B**) but not wortmannin (**A**) prevented MCF-7 cells from **II.2d** induced cell death. * Statistical significance with values of *p* < 0.05; ** statistical significance with values of *p* < 0.01.

Conversely, preincubation with wortmannin (Figure 9A) could not prevent cell death caused by compound II.2d, whereas preincubation with Z-VAD-FMK prevented it (Figure 9B). Thus, compound II.2d seems to act through a caspase-dependent mechanism.

**Figure 10:**
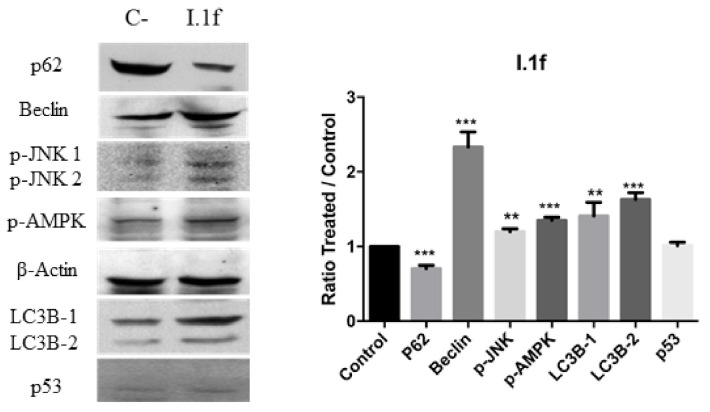
Western blot analysis of autophagy markers SQSTM1/p62, Beclin-1, p-JNKm p-AMPK, LC3B and p53 of MCF-7 cell culture treated with I.1f. ** statistical significance with values of *p* < 0.01; *** statistical significance with values of *p* < 0.001.

**Figure 11:**
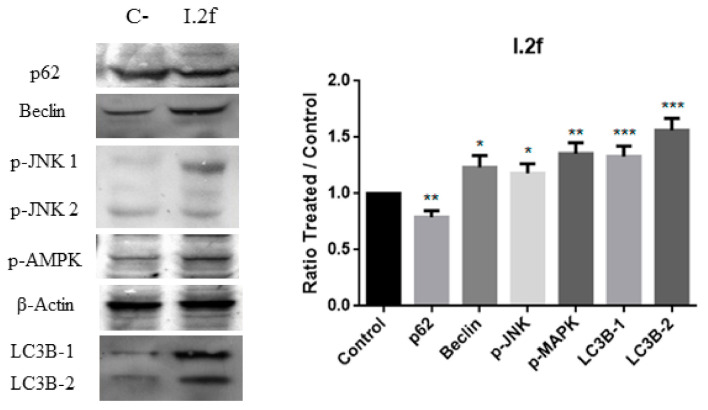
Western blot analysis of autophagy markers SQSTM1/p62, Beclin-1, p-JNKm p-AMPK and LC3B in MCF-7 cell culture treated with I.2f. * Statistical significance with values of *p* < 0.05; ** statistical significance with values of *p* < 0.01; *** statistical significance with values of *p* < 0.001.

To further confirm the programmed cell death pathway, the expression levels of several markers of autophagy [39] and apoptosis [40] were evaluated by Western blot. Beclin-1 and LC3B were determined, and autophagic flux was also assessed by testing SQSTM1/p62. As shown in Figure 10 and Figure 11, increased levels of Beclin-1 and LC3B were detected in MCF-7 cells when treated with I.1f (80 μM during 48 h) and I.2f (30 μM for 24 h), indicating autophagy. In addition, the autophagic flux, SQSTM1/p62, was downregulated confirming the autophagy process. Phosphorylation of AMPK and JNK was also studied because they have shown to be implicated in autophagy-mediated cell death [27]. As expected, both compounds induced JNK and AMPK phosphorylation.

**Figure 12:**
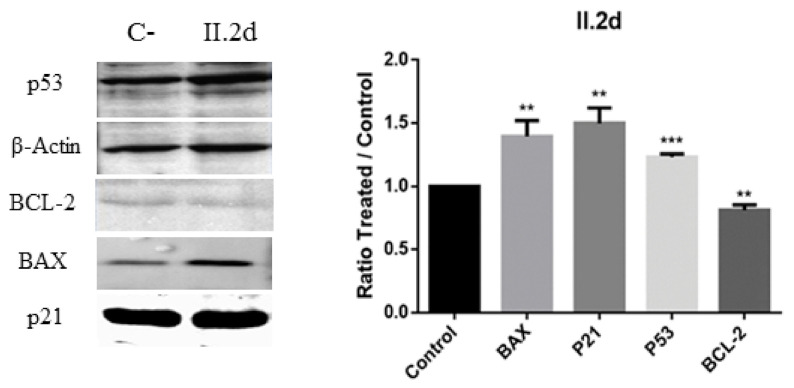
Western blot analysis of apoptosis markers BAX, p21, p53 and Bcl-2 in MCF-7 cell culture treated with II.2d. ** statistical significance with values of *p* < 0.01; *** statistical significance with values of *p* < 0.001.

Bax is an important proapoptotic protein implicated in apoptosis induction. On the contrary, Bcl-2 is an important antiapoptotic protein that suppresses apoptosis. The balance between Bax and Bcl-2 is very important for judgmental cell apoptosis [41]. The expression of apoptotic cell death markers was studied in MCF-7 cells after exposure to II.2d (Figure 12). Significantly increased levels of BAX, p21, p53 and decreased levels of Bcl-2 were detected, thus indicating apoptosis.

## 4. Conclusions

Forty-one new amides and phosphoramidates bearing selenocyanate and diselenide scaffolds were prescreened against three tumor cell lines at two doses. Twelve compounds were selected and tested against a panel of five tumor cell lines and two nonmalignant cell lines. The breast tumor cell line MCF-7 was the most sensitive, with six compounds showing GI_50_ values < 1 µM (I.1f, I.2b, I.2e, I.2f, II.2b and II.2d). These compounds were approximately three-fold more potent than cisplatin, a reference drug used clinically. Additionally, in vitro radical scavenging studies have shown that only series I compounds (amide derivatives) present antioxidant activity. Surprisingly, no phosphoramidate showed this effect. No correlation was found for either structure- or cytotoxicity-antioxidant activity, except for compound I.2f. Moreover, compounds I.1f, I.2f and II.2d showed the highest SI values when comparing the cytotoxic activity against 184B5 nonmalignant cell lines and were selected for further biological studies. Flow cytometry studies suggested that amides I.1f and I.2f induced cell cycle arrest at the S phase and cell death in a time- and dose-dependent manner and cell death through an autophagy process. Meanwhile, phosphoramidate II.2d flow cytometric studies showed an early caspase-dependent cell death at low doses and cell cycle arrest at G_0_/G_1_ phase at a 5 µM dose. However, as the dose increases, the number of subdiploid cells in the SubG1 phase also increases significantly. Both autophagy processes caused by I.1f and I.2f and apoptosis induction caused by II.2d were confirmed by Western blot analysis.

## Figures and Tables

**Table 1:** Average ± SEM values for GI_50_, TGI and LC_50_ parameters in MCF-7, CCRF-CEM, HT-29, HTB54 and PC-3 cell lines.

Comp	Cell Lines														
	MCF-7			CCRF-CEM			HT-29			HTB54			PC-3		
	GI_50_^a^	TGI ^b^	LC_50_^c^	GI_50_^a^	TGI ^b^	LC_50_^c^	GI_50_^a^	TGI ^b^	LC_50_^c^	GI_50_^a^	TGI ^b^	LC_50_^c^	GI_50_^a^	TGI ^b^	LC_50_^c^
I.1b	4.6 ± 0.5	>100	>100	9.0 ± 1.6	72.6 ± 1.1	>100	3.0 ± 1.8	7.7 ± 0.6	>100	>100	>100	>100	7.7 ± 2.2	>100	>100
I.1e	7.4 ± 1.0	40.1 ± 0.1	73.4 ± 0.1	6.5 ± 1.5	33.8 ± 2.0	67.0 ± 2.0	16.8 ± 2.5	44.6 ± 3.1	72.3 ± 1.8	16.9 ± 3.1	44.8 ± 2.3	72.8 ± 1.4	2.5 ± 0.2	28.5 ± 1.9	64.9 ± 1.1
I.1f	0.1 ± 0.0	13.0 ± 0.3	54.6 ± 0.7	4.7 ± 0.7	31.7 ± 1.8	79.2 ± 4.1	0.1 ± 0.0	5.7 ± 0.9	>100	25.1 ± 3.1	50.2 ± 2.4	75.4 ± 1.7	1.2 ± 1.5	32.9 ± 3.1	65.0 ± 0.6
I.1h	18.0 ± 2.3	47.3 ± 2.4	76.6 ± 2.5	4.1 ± 2.4	36.1 ± 1.4	72.8 ± 2.9	17.4 ± 1.9	45.8 ± 1.2	74.3 ± 0.7	20.8 ± 2.5	48.6 ± 1.8	76.3 ± 3.6	2.3 ± 0.3	27.6 ± 2.8	65.6 ± 4.1
I.2b	0.5 ± 0.9	51.8 ± 2.5	>100	8.7 ± 1.1	>100	>100	16.5 ± 3.6	>100	>100	>100	>100	>100	>100	>100	>100
I.2e	0.6 ± 0.1	54.4 ± 2.7	>100	31.6 ± 0.4	>100	>100	0.8 ± 0.1	58.2 ± 6.4	>100	>100	>100	>100	>100	>100	>100
I.2f	0.9 ± 0.0	6.2 ± 0.4	42.2 ± 0.8	7.3 ± 0.5	>100	>100	0.6 ± 0.4	79.2 ± 0.3	>100	29.8 ± 1.5	57.1 ± 3.3	84.4 ± 5.2	28.5 ± 0.7	53.1 ± 0.6	77.7 ± 0.6
I.2h	7.4 ± 1.9	50.4 ± 1.3	94.9 ± 4.7	21.8 ± 3.1	>100	>100	42.7 ± 1.2	>100	>100	>100	>100	>100	>100	>100	>100
II.1b	4.3 ± 0.6	8.8 ± 0.1	56.9 ± 5.4	2.0 ± 0.1	3.9 ± 0.4	8.0 ± 2.8	20.0 ± 3.1	47.4 ± 2.0	74.9 ± 0.6	7.2 ± 3.2	37.1 ± 3.2	76.0 ± 4.2	17.1 ± 1.7	46.9 ± 2.3	76.6 ± 2.5
II.1d	2.3 ± 0.2	4.2 ± 0.3	8.8 ± 0.8	0.4 ± 0.2	1.7 ± 0.2	3.4 ± 0.2	1.7 ± 0.1	3.0 ± 0.1	4.3 ± 0.1	5.9 ± 0.3	7.7 ± 0.2	9.4 ± 0.1	6.4 ± 0.2	8.6 ± 0.2	35.1 ± 4.9
II.2b	0.6 ± 0.2	5.0 ± 1.0	9.1 ± 0.7	1.6 ± 0.2	3.2 ± 0.2	4.8 ± 0.3	2.0 ± 0.3	3.5 ± 0.1	5.0 ± 0.1	6.2 ± 0.2	8.6 ± 0.4	34.6 ± 0.4	6.4 ± 0.2	9.3 ± 0.1	49.0 ± 0.7
II.2d	0.9 ± 0.1	4.1 ± 0.1	8.4 ± 0.1	0.2 ± 0.0	0.5 ± 0.0	0.9 ± 0.1	5.0 ± 0.8	7.3 ± 0.2	9.5 ± 0.4	1.6 ± 0.4	3.2 ± 0.3	4.8 ± 0.2	1.9 ± 0.1	4.4 ± 0.1	99.9 ± 9.5
Cp ^d^	3.2	>100	>100	1.0	79.6	>100	7.9	>100	>100	9.6	32.7	50.0	5.0	50.1	>100

^a^ GI_50_, concentration that reduces growth by 50% compared to control. ^b^ TGI, concentration that completely inhibits cell growth. ^c^ LC_50_, concentration that kills 50% of cells, ^d^ Cp: cisplatin; the GI_50_, TGI and LC_50_ values for Cp were obtained from the DTP database of the NCI [32].

**Table 2:** Average ± SEM values for GI_50_, TGI and LC_50_ parameters in 184B5 and BEAS-2B cell lines and selectivity indexes (SI).

Comp.	Cell Lines							
	184B5			SI ^d^	BEAS-2B			SI ^d^
	GI_50_^a^	TGI ^b^	LC_50_^c^		GI_50_^a^	TGI ^b^	LC_50_^c^	
I.1b	7.08	>100	>100	1.57	1.11	42.78	>100	<0
I.1e	7.76	33.79	68.16	1.14	0.42	4.80	15.81	0.02
I.1f	6.59	43.95	92.30	82.38	10.66	41.79	72.91	0.42
I.1h	19.94	47.19	74.44	1.11	10.60	40.65	70.70	0.51
I.2b	0.89	>100	>100	1.53	>100	>100	>100	<0
I.2e	7.13	>100	>100	20.97	>100	>100	>100	<0
I.2f	16.59	>100	>100	17.84	20.76	46.96	73.15	0.70
I.2h	36,1	>100	>100	5.66	8.47	67.32	>100	<0
II.1b	5.75	7.20	8.65	1.36	6.48	41.33	>100	0.84
II.1d	5.45	7.01	8.58	2.38	5.43	6.98	8.54	0.92
II.2b	5.44	6.94	8.44	7.56	5.22	6.80	8.38	0.84
II.2d	5.70	7.31	8.93	6.33	4.65	6.59	8.30	2.85

^a^ GI_50_, concentration that reduces growth by 50% compared to control. ^b^ TGI, concentration that completely inhibits cell growth. ^c^ LC_50_, concentration that kills 50% of cells. ^d^ Selectivity index (SI) = GI_50_ (184B5)/ GI_50_ (MCF-7) and GI_50_ (BEAS-2B)/GI_50_ (HTB-54).

## Data Availability

The data underlying this article will be shared upon reasonable request to the corresponding author.
